# Spectral data of refractive index and extinction coefficient for thin films of titanium group metals used for fabrication of optical microstructures

**DOI:** 10.1016/j.dib.2019.104903

**Published:** 2019-11-28

**Authors:** Dmitrij A. Belousov, Vadim S. Terent'ev, Evgeny V. Spesivtsev, Victor P. Korolkov

**Affiliations:** a Institute of Automation and Electrometry of the SB RAS, Koptyuga Avenue, 1, Novosibirsk, 630090, Russian Federation; b Rzhanov Institute of Semiconductor Physics of SB RAS, Lavrentieva Avenue, 13, Novosibirsk, 630090, Russian Federation

**Keywords:** Refractive index, Extinction coefficient, Hafnium, Zirconium, Titanium, Thin metal films, Ellipsometry, Thin film characterization

## Abstract

In this data paper we share the information on refractive index and extinction coefficient of metallic films of the titanium group (Ti, Zr, Hf), measured by ellipsometry in the wavelength range of 300–1100 nm. The presented data can be used to indirectly measure the thickness of metal films when they are sputtered onto a substrate, using the measured data on transmission and reflection coefficients depending on the wavelength of the probe beam, as well as to calculate the energy characteristics of diffraction gratings, formed on the surface of these films, under rigorous electromagnetic theory. The data were used in the research article “Increasing the spatial resolution of direct laser writing of diffractive structures on thin films of titanium group metals” [1].

**Table:** Specifications Table

Subject	Materials Science
Specific subject area	Surfaces, Coatings and Films
Type of data	Graphs, tables
How data were acquired	The data were obtained by the method of spectral ellipsometry using the device “ELLIPS-1991”, developed in Rzhanov Institute of Semiconductor Physics of SB RAS.
Data format	Raw
Parameters for data collection	Thin metal films were deposited onto a fused silica substrate by a magnetron sputtering technique. The measurements were made on Ti and Zr films with 1% transmission and Hf film with 0.13% transmission at 650 nm wavelength.
Description of data collection	Spectral range of measurement 300–1100 nm, beam diameter −1 mm.
Data source location	Rzhanov Institute of Semiconductor Physics of SB RAS, Novosibirsk, Russian Federation
Data accessibility	With the article as separate files
Related research article	V.P. Korolkov, A.G. Sedukhin, D.A. Belousov, R.V. Shimansky, V.N. Khomutov, S.L. Mikerin, E.V. Spesivtsev, R.I. Kutz, Increasing the spatial resolution of direct laser writing of diffractive structures on thin films of titanium group metals, Proc. SPIE 11030, Holography: Adv. and Mod. Trends VI 11030 (2019) 110300A. https://doi.org/10.1117/12.2520978 [1]

**Table:** 

**Value of the Data** • The data can be used to calculate reflection and transmission of the light illuminated diffractive micro/nano-structures formed on the surface of the Ti, Zr or Hf films when using rigorous electromagnetic theory. • Optical parameters of the metal films are very important at fabrication of micro/nanostructures on their surface. • This data can be used for fast estimation of the thickness of metal films when they are sputtered onto the substrate as shown in Ref. [1]. • The spectral dependences of refractive index and extinction coefficient for hafnium in 300–1100 nm wavelength range were obtained for the first time by our knowledge.

## 1 Data

**Fig. 1:**
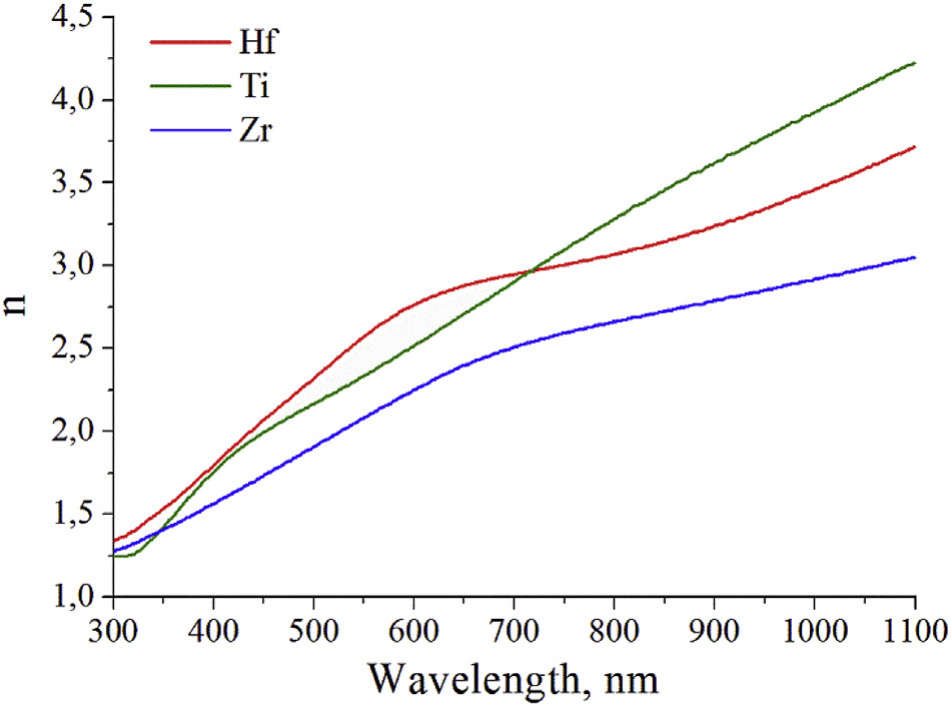
Spectral dependence of refractive index for thin films of Hf, Ti, and Zr.

**Fig. 2:**
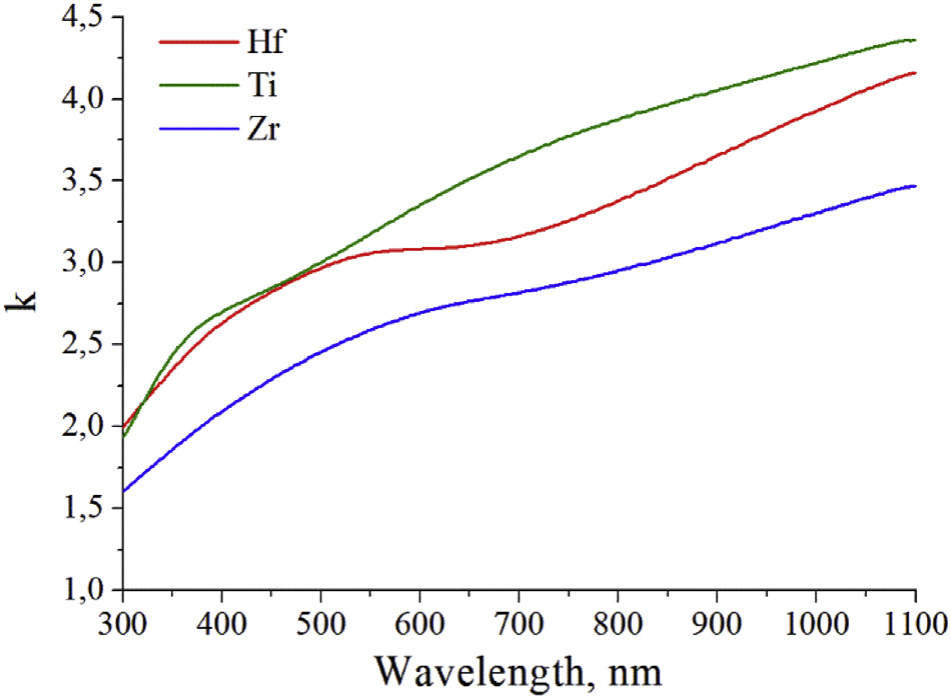
Spectral dependence of extinction coefficient for thin films of Hf, Ti, and Zr.

Here we show measured data of spectral dependence of refractive index (Fig. 1) and extinction coefficient (Fig. 2) for thin films of Hf, Ti, and Zr. The zirconium film has minimal values of extinction coefficient and refractive index in spectral range of 350–1100 nm.

## 2 Experimental design, materials, and methods

The data were obtained by the method of spectral ellipsometry using the device “ELLIPS-1991”, which uses the original static dual-channel scheme developed and patented in the ISP SB RAS [2]. As a light source, this device uses a 75 W xenon arc lamp, and the spectral measurement range is 300–1100 nm with a resolution of 2 nm. The probe beam diameter onto sample surface has a size of 1 mm. The refractive indices n and the extinction coefficients k of the metal films were calculated from the measured ellipsometric spectrums using the software of this ellipsometer.

The measurements were made on Ti and Zr films with 1% transmission and Hf film with 0.13% transmission measured at 650 nm wavelength. The studied thin metal films were deposited onto fused silica substrates by a vacuum magnetron sputtering technique. At that, targets of Ti, Zr and Hf with the purity of the materials of not less than 99.9% were used. The metal films were sputtered onto the hot surface of the substrate. The cleaned substrate was placed in a vacuum chamber and heated for 30 minutes in a vacuum of 10^−1^ mmHg at 200^о^ С. After warming up, the chamber was pumped out to a pressure of 5 × 10^−5^ mmHg. The substrate was located at a distance of 25 mm from the surface of the magnetron target (Kurt j. Lesker TORUS TM3 - 3”) above its center. The power supply time mode of the magnetrons was low-frequency voltage pulses with a duty cycle of 10, following with the frequency of 20–30 kHz. Using the frequency selection, the average discharge current was selected (I = 0.3 A). The magnetron discharge was stabilized by voltage (U = 1 kV) to maximize the energy of the sputtered particles. Argon was used as a buffer gas. Its pressure during the sputtering was 10^−3^ mmHg, to achieve free (hammerless with the atoms of the buffer gas) deposition of sputtered particles on the surface of the substrate. The buffer gas was supplied directly to the target of the magnetron. The characteristic sputtering rate was about 4 nm/s.

**Fig. 3:**
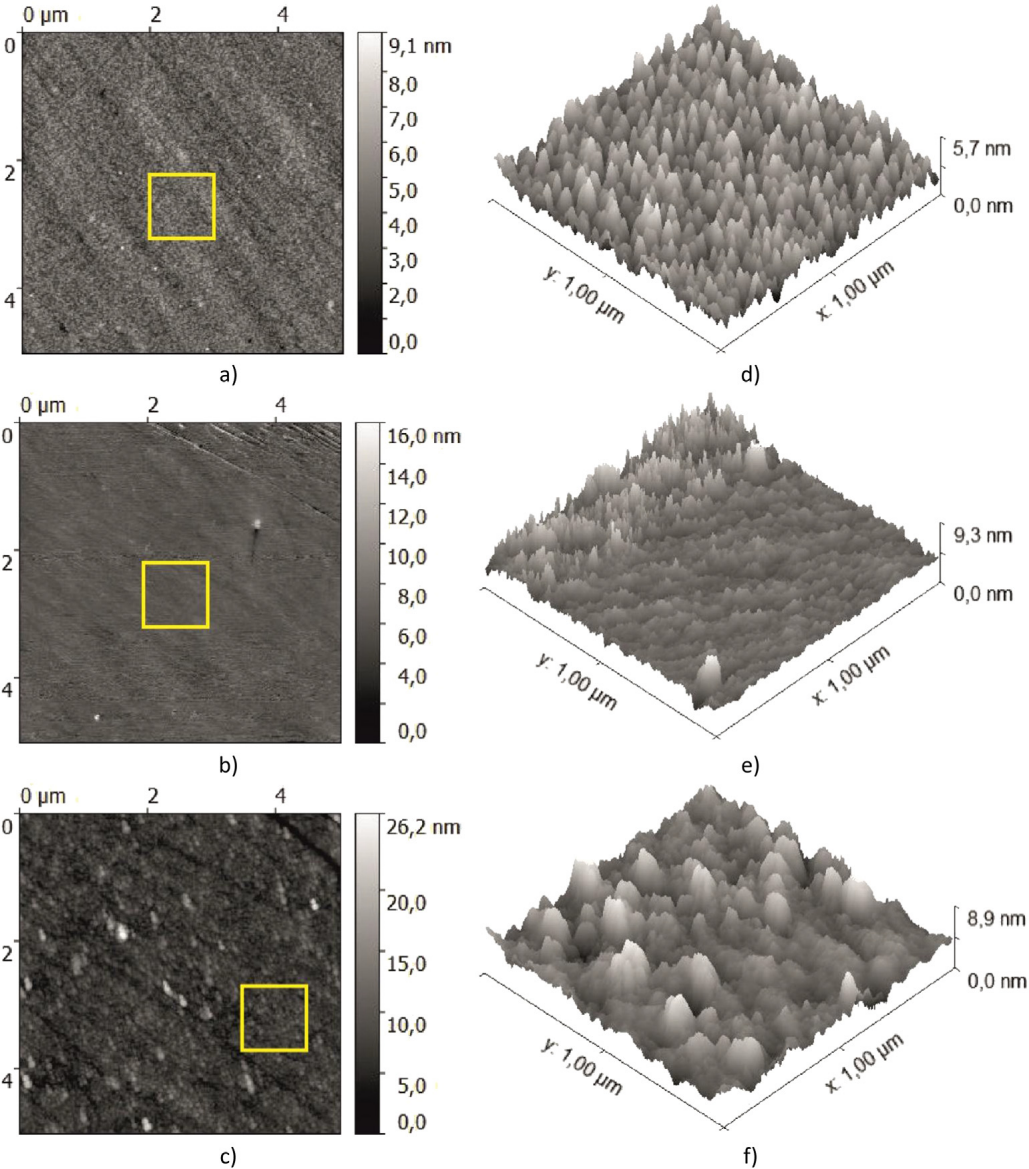
AFM-images of the investigated metal films: 2D - maps of the 5 × 5 μm area for Hf (a), Ti (b), and Zr (c), 3D-maps of the selected (yellow square) area with size of 1 × 1 μm for Hf (d), Ti (e), and Zr (f).

Using the Ntegra by NT-MDT atomic-force microscope (AFM), images of the surfaces of the investigated metal films were obtained (Fig. 3a–f). The obtained AFM-images show that the most homogeneous is the hafnium film sample. The Zr film has a microcrystalline structure. Diagonal fringes on the images of areas 5х5 μm are explained by surface imperfections of fused silica microscope slides with 1-mm thickness used as substrates for the film sputtering.
